# A Rare Observation of Brachymetacarpia and Brachymetatarsia in a Patient with Primary Idiopathic Hypoparathyroidism

**DOI:** 10.1155/2023/4149677

**Published:** 2023-02-28

**Authors:** Parackrama Karunathilake, Arun Rajaratnam, W. K. S. Kularatne

**Affiliations:** National Hospital Kandy, Kandy 20000, Sri Lanka

## Abstract

Brachymetacarpia and brachymetatarsia are unique clinical entities associated with numerous rare conditions. Primary hypoparathyroidism is distinct from pseudohypoparathyroidism and pseudopseudohypoparathyroidism by lacking skeletal changes such as short metacarpals or metatarsals. Here, we present a case of a 64-year-old patient with brachymetacarpia and brachymetatarsia presented with hypocalcemic symptoms and signs, bilateral cataracts, and basal ganglia calcifications, subsequently diagnosed with idiopathic primary hypoparathyroidism. This is a rare case describing such an infrequent observation of brachymetacarpia and brachymetatarsia in primary idiopathic hypoparathyroidism.

## 1. Introduction

Brachydactyly is characterized by the shortening of digits in diverse congenital or acquired disorders, either as an isolated malformation or with other skeletal manifestations [1]. Brachymetacarpia and brachymetatarsia are rare congenital differences of the extremities defined by the shortening of metacarpals and metatarsals, respectively, due to premature fusion of the growth plate or due to pathology in the bone itself [1, 2]. The cause may be a syndrome acquired from diseases in childhood or idiopathy [1, 3]. Shortening of metacarpals is a useful diagnostic marker in patients with pseudohypoparathyroidism and is rarely reported in cases of idiopathic primary hypoparathyroidism [4]. Here, we report an uncommon case of primary idiopathic hypoparathyroidism with brachymetacarpia and brachymetatarsia.

## 2. Case Presentation

**Figure 1:**
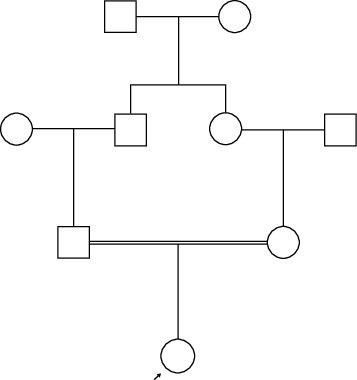
Genogram illustrating the consanguinity.

A 64-year-old, postmenopausal Sinhalese woman with stable chronic plaque psoriasis for 10 years presented to a tertiary care center in Sri Lanka with insidious onset, nonprogressive, intermittent numbness in the distal aspects of bilateral upper, and lower extremities for two weeks. She described a tingling sensation over her distal upper and lower limbs and a cramping sensation in her calves and arms. She did not have any peri-oral numbness, carpopedal spasms, dyspnoea, palpitations, changes in mood, amnesia, or seizures. Direct questioning revealed that she had occasional carpopedal spasms in her thirties and had been prescribed calcium supplements to control those. She also stated that her parents are second cousins (Figure 1). She also gave a history of taking medications for childhood epilepsy for less than 5 years. None of her first-degree relatives had similar symptoms, syndromic appearances, or skeletal changes. She had an age-appropriate developmental history, an uncomplicated menstrual history, and no history of trauma or infection to her limbs. She was a mother of three children and did not have any medical ailments complicating the pregnancy.

**Figure 2:**
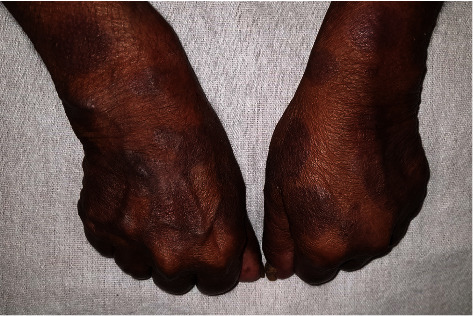
Short fourth and fifth metacarpals.

**Figure 3:**
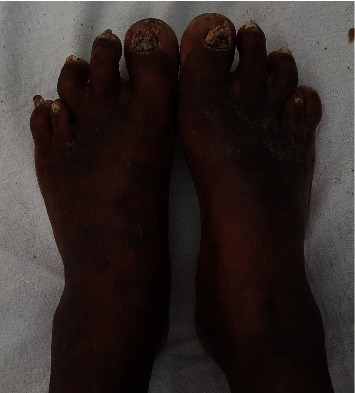
Short fourth metatarsals.

The examination revealed normal hemodynamic and respiratory parameters without objective sensory loss or spasms. Chvostek's and Trousseau's signs were negative. She was thin built with a body mass index of 21.64 kg/m^2^ (weight—45.5 kg, height—1.45 m), mildly pigmented with healed psoriatic plaques. Her eye examination revealed bilateral cataracts; she was noticed to have bilateral short fourth and fifth metacarpals (Figure 2) and short fourth metatarsals (Figure 3). We visually confirmed that her siblings or her daughter were not having similar changes in their digits.

**Figure 4:**
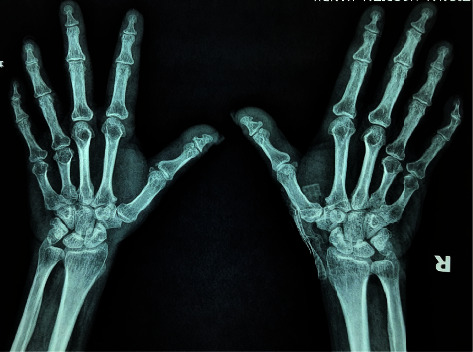
X-ray of bilateral hands showing brachymetacarpia.

**Figure 5:**
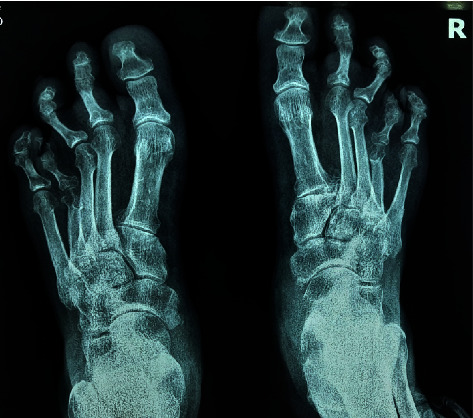
X-ray of bilateral feet showing brachymetatarsia.

**Figure 6:**
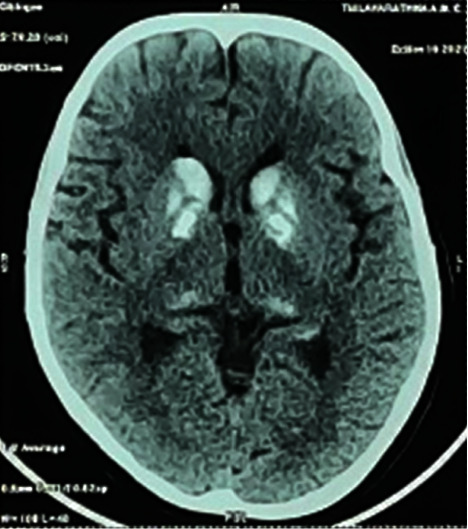
NCCT brain showing basal ganglia calcifications.

A summary of her blood investigations is shown in Table 1. Her hand and feet radiographs confirmed brachymetacarpia and brachymetatarsia (Figure 4 and 5), and her noncontrast CT scan of the brain showed basal ganglia calcifications (Figure 6). A neck ultrasound scan showed the thyroid gland of normal size and echotexture (right lobe—12 × 8 mm; left lobe—9 × 9 mm), without any thyroid nodules, enlarged parathyroid glands, or cervical lymphadenopathy. The electrocardiogram showed a corrected QT interval of 500 milliseconds.

The diagnosis of hypoparathyroidism with subclinical hypothyroidism was made, and she was started on an intravenous calcium gluconate infusion at 0.5 ml/kg/hour with continuous ECG monitoring. Then, her serum ionized calcium improved gradually to 1.18 and then to 1.23 mmol/L, and she complained of lesser subjective numbness. With the improvement of symptoms, the treatment regimen was switched to oral calcium carbonate 1500 mg (3 tablets) three times a day, oral calcitriol 0.25 ug three times a day, and oral magnesium sulfate 800 mg three times daily, and she was discharged after 6 days of inward admission. At the clinic review in 1 month, her hypocalcemic symptoms had improved, and the serum ionized calcium levels were within the normal limits.

## 3. Discussion

Brachymetacarpia and brachymetatarsia are rare anatomical malformations of the extremities defined by the shortening of metacarpals and metatarsals, occurring more commonly in females than in males [2, 5]. Brachymetacarpia occurs more commonly in the third, fourth, and fifth fingers, whereas brachymetatarsia commonly affects the first and fourth metatarsals [2, 4]. Both these conditions occur due to premature fusion of the growth plate or pathology in the bone itself, and the etiology can be either congenital or acquired [2]. Sporadic mutations and other congenital deformities of the limbs such as syndactyly, polydactyly, cleft hand symbrachydactyly, accessory navicular, and deformities of the nail and nail bed are possible associations of these conditions [2]. The incidence of brachymetatarsia ranges from 0.02% to 0.05%, and the incidence of brachymetacarpia is less than 1 in 1000. The occurrence of bilateral brachymetacarpia and brachymetatarsia in a single patient is extremely rare [1]. The causative gene defects have been identified for the majority of isolated brachydactyly and some syndromic forms of brachydactyly. In isolated brachydactyly, the inheritance is mostly autosomal dominant with variable expressivity and penetrance [6].

Brachymetacarpia and brachymetatarsia can be seen in numerous conditions, including Klinefelter syndrome, Russel–Silver syndrome, Turner syndrome, De Lange syndrome, Down syndrome, Taybi syndrome, and achondroplasia [3, 7], and they typically present in pseudohypoparathyroidism which occurs due to end-organ resistance [8]. The shortening of the fourth metacarpal is especially seen in Turner's syndrome and dyschondrosteosis [3]. Shortening of metacarpals is a useful diagnostic marker in patients with pseudohypoparathyroidism type Ia with Albright's hereditary osteodystrophy (AHO) phenotype or pseudopseudohypoparathyroidism [4]. Very rarely short metacarpals can be seen in patients with primary idiopathic hypoparathyroidism, as reported in very few cases in the literature [4, 9, 10]. Furthermore, the simultaneous existence of both brachymetacarpia and brachymetatarsia in primary idiopathic hypoparathyroidism is reported in even fewer cases [11]. Nayak and Samal have reported a case of short fourth metacarpal and short fourth metatarsal bilaterally in a patient with primary hypoparathyroidism; however, our case has its unique feature different from this previous case because our patient had bilateral short fourth and fifth metacarpals with short fourth metatarsals.

The complaints of patients with brachymetacarpia are impaired hand function and cosmetic concerns. Impaired hand function possibly occurs due to the relative lengthening of the corresponding muscle-tendon unit resulting in reduced force generated from the contraction of these muscles, which can manifest as weakness of hand function. Shortening any metatarsal may also stretch the transverse metatarsal ligament, consequently leading to altered forefoot contact with the ground and excessive pressure. Hence, in brachymetatarsia, the metatarsal shortening can lead to suboptimal muscles, leading to fatigue pain in the leg and foot [2]. Nevertheless, our patient did not complain of these symptoms due to brachymetacarpia or brachymetatarsia.

It is noteworthy that this elderly lady did not have any other syndromic phenotypes of the other genetic conditions that can cause brachymetatarsia and brachymetacarpia, despite her background history of the consanguinity of her parents. Also, none of her other siblings or relatives had similar skeletal abnormalities. The unavailability of advanced genetic studies in Sri Lanka limited further investigation of our case. It is previously known that hypoparathyroidism is distinct from pseudohypoparathyroidism and pseudo-pseudohypoparathyroidism by the absence of metacarpal or metatarsal abnormalities. The reported case and a few others shed new knowledge, revealing that primary idiopathic hypoparathyroidism rarely produces such skeletal abnormalities [4, 9–11].

## 4. Conclusion

Brachymetacarpia and brachymetatarsia are rare physical signs in patients with pseudohypoparathyroidism and pseudopseudohypoparathyroidism. However, these can be uncommonly present in primary idiopathic hypoparathyroidism as well. A relevant history covering past trauma, infection, familial diseases, a targeted clinical examination, and performing the parathyroid-calcium axis's radiological, genetic, and biochemical investigation may illuminate the underlying pathological associations.

## Figures and Tables

**Table 1:** Summary of the investigation results.

Investigation	Value	Normal range
Serum ionized calcium	0.54 mmol/L	2.2–2.6 mmol/L
Serum phosphate	0.37 mmol/L	0.97–1.45 mmol/L
Serum magnesium	0.69 mmol/L	0.85–1.10 mmol/L
Serum albumin	3.5 g/dL	3.2–5.4 g/dL
Vitamin D level	13.07 ng/mL	10–29 ng/mL
Parathyroid hormone level	4.22 pg/mL	15–35 pg/mL

Urine calcium/creatinine ratio	0.46	<0.14

White cell count	10.9 × 10^3^/*μ*L	4.5 × 10^3^–11 × 10^3^/*μ*L
Neutrophils %	66%	45–68%
Hemoglobin	10.6 g/dL	11.6–15.0 g/dL
Mean corpuscular volume	95.5 fL	86–100 fL
Platelet count	478 × 10^3^/*μ*L	150–450/*μ*L

ESR	100 mm/1^st^ hour	<30 mm/1^st^ hour
C-reactive protein level	23 mg/dL	<10 mg/dL

Serum sodium	137 mmol/L	135–145 mmol/L
Serum potassium	3.5 mmol/L	3.5–5.0 mmol/L

Fasting blood sugar	118 mg/dL	<126 mmol/L
Aspartate transaminase	40 U/L	40 U/L
Alanine transaminase	22 U/L	25 U/L
Alkaline phosphatase	92.32 U/L	44–147 U/L
Serum creatinine	79.98 *μ*mol/L	53–97 *μ*mol/L
Blood urea	6.3 mmol/L	2.1–8.5 mmol/L

TSH	9.87 mIU/L	0.5–5 mIU/L
Free T4	16.1 pmol/L	12–30 pmol/L

## Data Availability

All the data are stored in a password-protected computer, and hard copies are also under the corresponding author's custody. Data can be seen by request to the corresponding author.
